# Reversible amorphization and the catalytically active state of crystalline Co_3_O_4_ during oxygen evolution

**DOI:** 10.1038/ncomms9625

**Published:** 2015-10-12

**Authors:** Arno Bergmann, Elias Martinez-Moreno, Detre Teschner, Petko Chernev, Manuel Gliech, Jorge Ferreira de Araújo, Tobias Reier, Holger Dau, Peter Strasser

**Affiliations:** 1 Department of Chemistry, Chemical and Materials Engineering Division, Electrochemical Energy, Catalysis and Materials Science Laboratory, Technische Universität Berlin, Straße des 17. Juni 124, 10623 Berlin, Germany; 2 Department of Physics, Freie Universität Berlin, Arnimallee 14, 14195 Berlin, Germany; 3 Department of Inorganic Chemistry, Fritz-Haber-Institute of the Max-Planck-Society, Faradayweg 4-6, 14195 Berlin, Germany; 4 Ertl Center for Electrochemistry and Catalysis, Gwangju Institute of Science and Technology, Gwangju 500-712, South Korea

## Abstract

Water splitting catalysed by earth-abundant materials is pivotal for global-scale production of non-fossil fuels, yet our understanding of the active catalyst structure and reactivity is still insufficient. Here we report on the structurally reversible evolution of crystalline Co_3_O_4_ electrocatalysts during oxygen evolution reaction identified using advanced *in situ* X-ray techniques. At electrode potentials facilitating oxygen evolution, a sub-nanometre shell of the Co_3_O_4_ is transformed into an X-ray amorphous CoO_*x*_(OH)_*y*_ which comprises di-μ-oxo-bridged Co^3+/4+^ ions. Unlike irreversible amorphizations, here, the formation of the catalytically-active layer is reversed by re-crystallization upon return to non-catalytic electrode conditions. The Co_3_O_4_ material thus combines the stability advantages of a controlled, stable crystalline material with high catalytic activity, thanks to the structural flexibility of its active amorphous oxides. We propose that crystalline oxides may be tailored for generating reactive amorphous surface layers at catalytic potentials, just to return to their stable crystalline state under rest conditions.

## Abstract (web summary)

Understanding of catalyst structure and reactivity is important for the development of water splitting catalysts. Here, the authors report reversible structural transformation of the near-surface of crystalline Co_3_O_4_ electrocatalysts to an amorphous CoO_x_(OH)_y_ during oxygen evolution.

## Graphical abstract

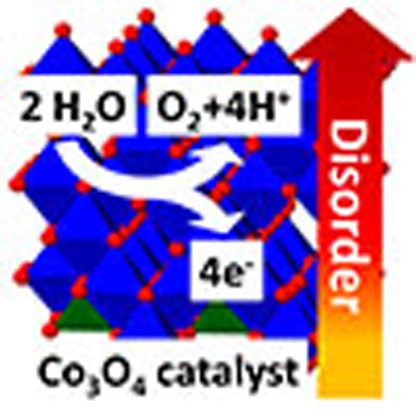

Tailoring active, stable and inexpensive electrocatalysts for water splitting and oxygen evolution reaction (OER) is a major issue for the utilization of hydrogen as a chemical storage of electrical energy from intermittent renewable power sources^1^,^2^,^3^,^4^. Optimal design of these electrocatalysts requires extended knowledge about the catalytically-active site and activity-determining structural properties. Structural analysis of electrocatalysts under electrochemical reaction conditions thus is of special importance and is approached in the present investigation.

There is an ongoing controversy on the importance of structural disorder to achieve high catalytic activity (for example, special coordination or oxidation state of metal ions, defects and vacancies in the bulk of an amorphous material or at its surface, sub-nanometre-sized catalyst domains, presence of super-active sites)^5^,^6^,^7^,^8^,^9^,^10^. High water splitting activities have been reported for single crystals, (poly)crystalline and X-ray amorphous electrocatalysts based on Ru and Ir oxides working in acidic electrolytes^11^,^12^,^13^,^14^,^15^,^16^,^17^ as well as perovskites and NiFe layered double hydroxides under alkaline reaction conditions^18^,^19^,^20^,^21^,^22^.

A well-known X-ray amorphous OER electrocatalyst is the so-called cobalt-phosphate catalyst, herein denoted as CoCat^2^,^23^,^24^,^25^,^26^,^27^,^28^,^29^,^30^, which is catalytically active in neutral phosphate-containing electrolyte and excels by self-repair properties^29^. The CoCat films consist of layer fragments of ∼12–14 octahedrally coordinated (mainly) Co^3+^ ions connected via di-μ_2/3_-oxo bridges^23^,^24^,^25^. The CoO_*x*_ fragment size depends on the deposition conditions where a lower domain size is beneficial for catalytic activity^26^, in line with findings for OER catalysis by iridium oxides^13^,^17^. The μ_2_-O(H) bridges at the edges of the catalyst domains are thought to be involved under OER catalysis^26^,^31^.

Various crystalline (double) perovskites containing Co ions exhibit water splitting activity in alkaline electrolyte^20^. Depending on the initial composition of lanthanides, an irreversible loss of crystallinity was detected after potential cycling covering water oxidation potentials^18^,^32^,^33^. Irreversible surface amorphization was shown also for other Co-containing and initially crystalline catalysts^6^,^7^,^34^. A popular example of a crystalline Co oxide is the Co_3_O_4_ spinel. It shares structural motifs with the CoCat because it consists of Co-deficient CoO_6_ Kagomé layers, which are connected via Co^3+^ O_h_ ions (O_h_, octahedral coordination by six oxygen atoms) and Co^2+^ T_d_ ions (T_d_, tetrahedral coordination by four oxygen atoms). The O^2−^ lattice is a so-called pseudo-cubic close-packed (ccp) arrangement^35^,^36^,^37^,^38^,^39^,^40^.

Although the existence of irreversible surface amorphization during OER has been well established, reversible structural changes of electrocatalysts during gas evolution have remained completely unaddressed to date. Detection of reversible structural changes requires *in situ* analysis methods as approached herein. Past investigations of the active state of Co oxide-based electrocatalysts have been largely limited to electrodeposited, thus mostly X-ray amorphous films^27^,^41^,^42^,^43^,^44^. These studies showed that Co oxide redox features lead to oxidation state changes^27^,^42^, whereas oxygen evolution in alkaline electrolyte is accompanied by changes in Raman features indicating changes of the local atomic structure^43^.

In contrast to alkaline conditions, the electrocatalysis of water splitting of noble-metal-free catalysts for OER under benign reaction conditions has been receiving growing attention^2^,^10^,^23^,^24^,^25^,^26^,^28^,^31^,^45^,^46^,^47^. However, the structural evolution of crystalline electrocatalysts in neutral electrolyte has remained poorly understood. This is unfortunate considering that crystalline transition metal oxides (as films or supported nanoparticles) are commonly prepared using chemical precipitation methods closely related to established large-scale industrial syntheses. This study aims to change that.

Using an array of *in situ* bulk-sensitive X-ray scattering and spectroscopic techniques, this work unravels the local and long-range structure of the active state of crystalline Co_3_O_4_ film catalysts in neutral phosphate-containing electrolyte, with special emphasis on structural similarities and differences between the active state of amorphous and crystalline electrocatalysts. We report on a previously overlooked reversible transition between a crystalline rest state and an active amorphous state of the shell of the crystallites, which could potentially aid in the future design of more active and stable water splitting catalysts. Based on our insights, we consider it feasible that crystalline oxides form structurally flexible and hence catalytically active amorphous shells, which, upon restoring rest conditions, reversibly transform back in their thermodynamically more stable crystalline state.

## Results

### Morphology and local structure

**Figure 1:**
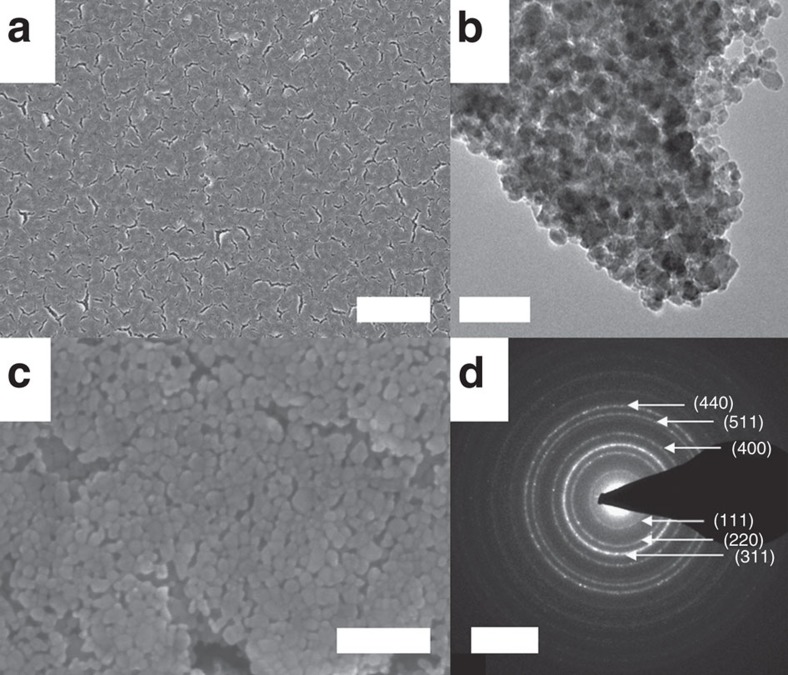
Structural and morphological characterization of Co_3_O_4_ films. Scanning electron micrographs in low (**a**) and high magnification (**c**), transmission electron micrograph (**b**) and selected area electron diffraction (SAED) pattern (**d**) of as-prepared Co_3_O_4_ films. Diffraction rings of Co_3_O_4_ are indexed in the SAED pattern. The scale bars represent 2 μm, 50 nm, 100 nm and 5 nm^−1^ in panel **a**,**b**,**c** and **d** respectively.

Figure 1 shows scanning and transmission electron micrographs of CoO_*x*_ films as well as selected area electron diffraction (SAED). The as-prepared films exhibited a homogeneous morphology with a certain porosity leading to increased electrolyte accessibility. The porosity of Co_3_O_4_ films was ∼0.46. (cf. Supplementary Fig. 1 for further details) Thus, OER was not limited to the outer surface of the CoO_*x*_ films. The metal loading was ∼960 nmol_Co_ cm^−2^ on glassy carbon (GC) substrate as determined by inductively coupled plasma optical emission spectroscopy (ICP-OES). The microstructure consisted of agglomerated crystallites with size of 5–10 nm. The SAED pattern showed a typical ring structure characteristic for polycrystalline materials and agrees well with the normal spinel structure of Co_3_O_4_.

### Electrochemical characterization in neutral electrolyte

**Figure 2:**
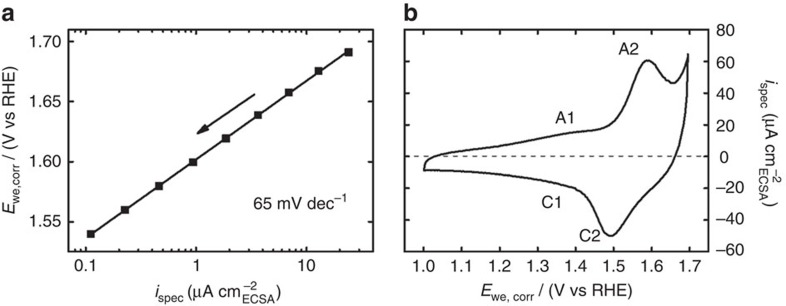
Electrochemical characterization of Co_3_O_4_ films. Tafel plot (**a**) and cyclic voltammogram (**b**) of Co_3_O_4_ films deposited on glassy carbon recorded in 0.1 M KP_i_ at pH 7. The Tafel plot was extracted from cathodic quasi-stationary potential-step rotating disc electrode experiments after equilibration for 4 min at each potential; the line corresponds to a Tafel slope and an exchange current density of 65 mV dec^−1^ and 2.26 × 10^−9^ mA cm^−2^_ECSA_, respectively. The cyclic voltammogram was recorded with a scan rate of 100 mV s^−1^ and shows a minor and major Co redox feature at ∼1.4 V (A1/C1) and ∼1.54 V (A2/C2), respectively. The capacitance-corrected reductive charge of the two redox features showed that only ∼1.8% of the Co ions change their oxidation state by one equivalent. Electrode potentials were corrected for Ohmic losses and are referred to reversible hydrogen electrode (RHE). The current was normalized using electrochemical surface area (ECSA) as determined by potentiostatic electrochemical impedance spectroscopy^14^. The electrochemical surface roughness was 26.4.

The electrocatalytic properties and Co oxide redox electrochemistry were investigated in N_2_-saturated 0.1 M KP_i_ at pH 7. Tafel plot and cyclic voltammogram are shown in Fig. 2. The electrochemical surface area was determined using potentiostatic impedance spectroscopy following ref. 14. (cf. Supplementary Fig. 2 for further information). Co_3_O_4_ films exhibited a pronounced redox feature at ∼1.54 V, which was preceded by a minor feature at a potential of ∼1.4 V. According to the Pourbaix diagram^48^ and a previous CoO_*x*_ study^27^, the redox feature at ∼1.54 V can be attributed to the Co^3+^/Co^4+^ redox transition, whereas the feature at about ∼1.4 V is consistent with a Co^2+^/Co^3+^ transition. Evaluation of the capacitance-corrected reductive charge of the two redox features showed that only ∼1.8% of the Co ions change their oxidation state by one equivalent during this cyclic voltammogram. Thus, we conclude that only Co ions from the surface participate in the Co oxide redox chemistry under this dynamic condition. The Co_3_O_4_ films exhibited a linear E—log(i) behaviour between 1.54 and 1.7 V with a Tafel slope of 65 mV dec^−1^ in N_2_-saturated electrolyte. The Tafel slope as determined from anodic polarization was slightly higher than that determined from cathodic quasi-stationary potential step experiment (cf. Supplementary Figs 3, 4 and 8). The Tafel slope of crystalline Co_3_O_4_ was found to be very similar compared with the one of electrodeposited Co oxide films, which exhibit ≥60 mV dec^−1^ in 0.1 M KP_i_.(refs 26, 30, 45). We note that catalytic activity and Co oxide redox behaviour showed very good stability (Supplementary Figs 3–9). Furthermore, OER did not influence morphology and Co loading (Supplementary Figs 10 and 11).

To verify electrocatalytical oxygen evolution of Co_3_O_4_ in neutral phosphate-containing electrolyte, we performed differential electrochemical mass-spectrometry (DEMS) experiments. Supplementary Fig. 12 shows a cyclic voltammogram of Co_3_O_4_ in the OER range and the corresponding ion current of *m*/*z*=32 and 44 as a measure of the evolution of oxygen and carbon dioxide, respectively. The onset potential of O_2_ evolution agreed well with the Co^3+^/Co^4+^ redox potential and O_2_ evolution followed the rise of the current density with electrode potential. Thus, we conclude that the Co_3_O_4_ films showed significant oxygen evolution activity above the Co^3+^/Co^4+^ redox couple at ∼1.54 V in neutral phosphate-containing electrolyte and exhibit typical Co oxide redox behaviour.

### Long-range order under electrochemical reaction conditions

**Figure 3:**
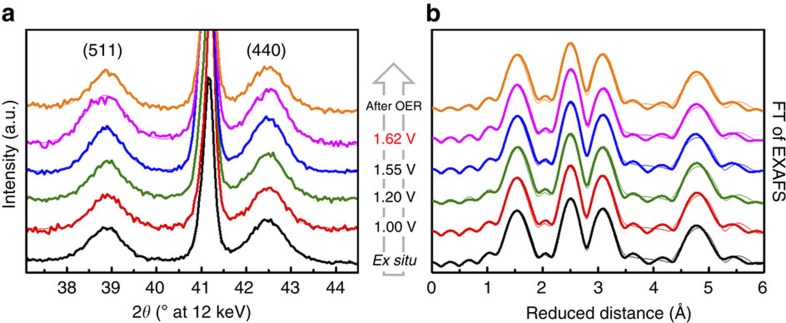
*In situ* structural characterization of Co_3_O_4_ films. *In situ* X-ray diffraction patterns (**a**) and Fourier-transforms (FT) of quasi-*in situ* EXAFS spectra collected at the Co *K*-edge (**b**) of Co_3_O_4_ catalyst films. Experimental data and fitted profiles are shown in bold and thin lines, respectively. The diffraction patterns were recorded using grazing-incident excitation at *α*=0.3° and 12 keV. The electrode potential was increased stepwise from +1.0 to +1.62 V versus reversible hydrogen electrode, the latter representing the catalytically active catalyst state, in 0.1 M KPi at pH 7 (cf. Fig. 2a and Supplementary Fig. 12). The state after OER is a dry state for which the electrode was removed from the electrolyte at +1.0 V rinsed with de-ionized water and dried in N_2_ flow. The Miller indices of selected Co_3_O_4_ reflections are indicated; the diffraction patterns were background corrected for better visualization. Fitting of the diffraction pattern was performed using pseudo-Voigt profiles. Samples for XAS were freeze-quenched under potential control using liquid N_2_ after 15 min at 1.62 V in 0.1 M KP_i_. Further details on data analysis are given in the caption of Supplementary Tables 1 and 2. See also Supplementary Figs 15 and 16.

**Figure 4:**
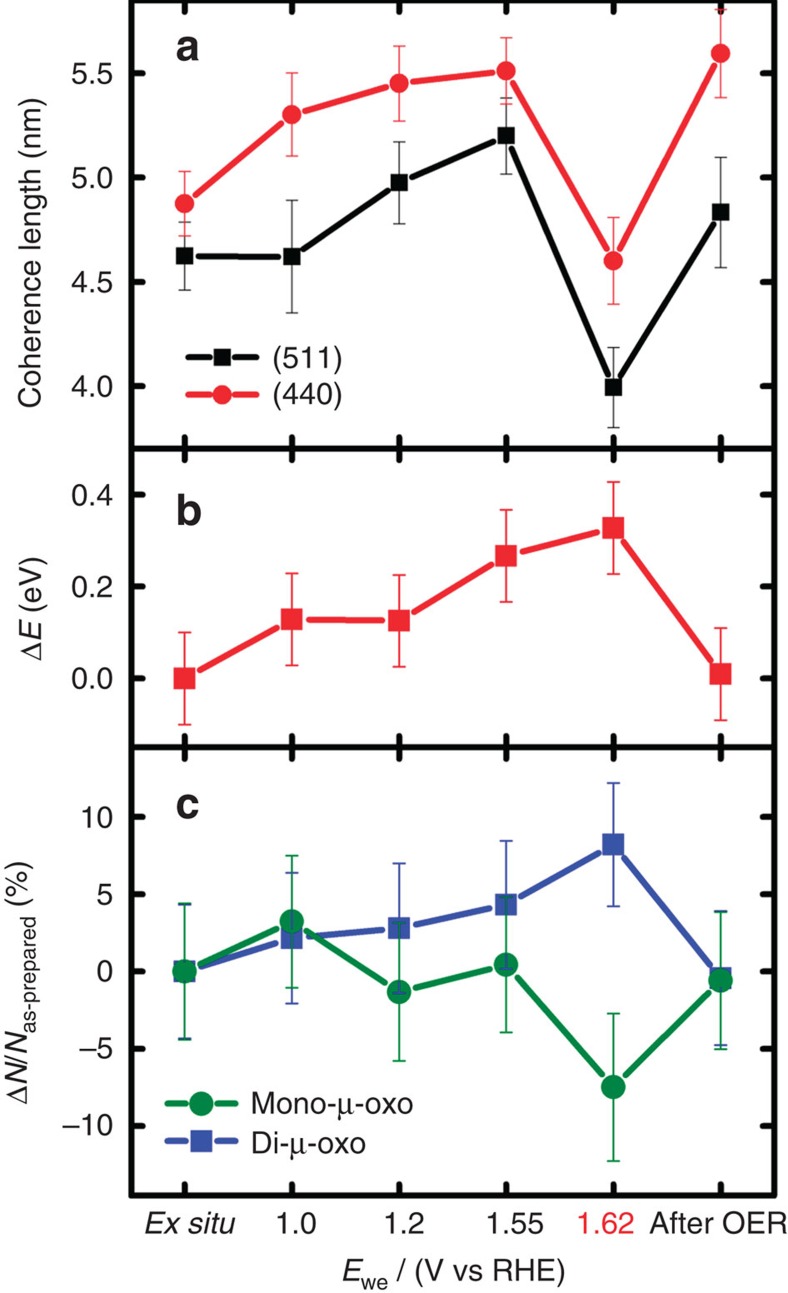
Quantitative analysis of *in situ* structural characterization of Co_3_O_4_ films. Structural coherence length of Co_3_O_4_ (**a**), shift of the Co X-ray absorption *K*-edge (**b**), change in EXAFS coordination number of di- and mono-μ-oxo bridges between Co ions (**c**) with respect to the as-prepared state. The state at +1.62 V corresponds to the catalytically active oxygen evolution state (cf. Fig. 2a and Supplementary Fig. 12). Electrode potentials in this particular experiment are not corrected for Ohmic losses but we note that Ohmic correction decreased the electrode potential at +1.62 V by less than ∼1 mV in the quasi-stationary experiment. The structural coherence length was calculated using the Scherrer equation from the integral breadth of the Co_3_O_4_(511) and (440) reflections^65^. A shape factor *k* of 0.89 was used. The error of the structural coherence. length was calculated from the estimated standard deviation of the fitted integral breadth. The likely error of the shift of the Co K edge was estimated to be ±0.1 eV. The coordination numbers were determined by simulations of k^3^-weighted EXAFS spectra. The error ranges of the EXAFS fit parameters were estimated from the covariance matrix of the fit and represent the 68% confidence intervals. Further details on data analysis are given in the caption of Supplementary Tables 1 and 2.

To examine atomic structure and crystallinity of Co_3_O_4_ films at different electrochemical potentials including the OER, we conducted *in situ* grazing-incident X-ray diffraction (GIXRD) experiments. Diffraction patterns of Co_3_O_4_ films deposited on Ti in the as-prepared state, at selected electrode potentials and after *in situ* characterization are shown in Supplementary Fig. 13 and Fig. 3a. Besides the reflections from the Ti substrate, Co_3_O_4_ (Space group: Fd-3m, PDF#00-042-1467) was the only detectable crystalline phase before, during, and after electrochemical reaction conditions. Thus, the catalyst film essentially retained its Co_3_O_4_ structure under the entire electrochemical potential range considered. However, a thorough analysis of the broadening of selected Co_3_O_4_ reflections (Fig. 4a and Supplementary Table 1) showed that the mean structural coherence length of the crystalline domains had changed. The structural coherence length increased after immersion into the electrolyte at +1.0 V and then increased further with more positive electrode potential. The largest structural coherence length was found at the onset of OER at +1.55 V, just anodic of the Co redox features (Fig. 2). As the electrode potential was increased further to +1.62 V, the structural coherence length of the Co_3_O_4_ domains decreased by almost 10 Å. In the dry state after OER, the structural coherence length recovered to the identical value as before OER at +1.2 V. Our observations can be plausibly explained by an initial irreversible growth of Co_3_O_4_ crystallites up to an electrode potential of +1.2 V, likely due to Ostwald ripening and/or coalescence^49^,^50^, combined with a reversible structural transformation of Co_3_O_4_ detectable at an electrode potential of +1.62 V, where catalytic oxygen evolution proceeded at elevated rates (cf. Supplementary Fig. 12). At the onset of OER (+1.55 V), the Co_3_O_4_ exhibited its largest structural coherence length. Thereafter, further increasing the oxygen evolution rate at more anodic electrode potentials resulted in a reversible structural transformation leading to a lower degree of crystallinity. This structural transformation can be explained with the formation of a CoO_*x*_ shell on the crystalline Co_3_O_4_ domains, which are in contact with the electrolyte. This CoO_*x*_ did not give rise to additional Bragg reflections because of its sub-nanometre size and is thus X-ray amorphous. On the reverse cathodic scan, upon leaving the potential range of sustained catalytic oxygen evolution associated with electrochemical reduction, the amorphous shell recrystallized to Co_3_O_4_. To our knowledge, this is the first time that such a reversible structural transformation of a shell on Co_3_O_4_ crystallites from crystalline to amorphous and back has been reported. We further emphasize that this unusual reversible amorphization of Co oxide appears to be linked to an elevated catalytic oxygen evolution rate.

### Local atomic structure under electrochemical reaction conditions

**Figure 5:**
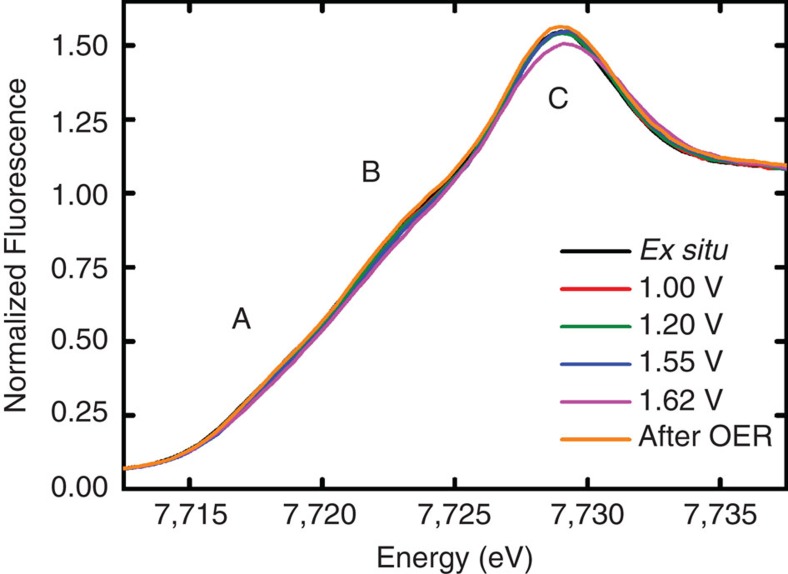
XANES profiles of Co_3_O_4_ under electrochemical conditions. Fluorescence-detected quasi-*in situ* XANES spectra of Co_3_O_4_ films in various catalyst states. For increasingly positive potentials, the Co *K*-edge absorption edge shifted reversibly to higher energies suggesting oxidation of 10–15% of the cobalt ions under stationary oxygen evolution conditions. Under OER conditions, the XANES profile exhibited an almost linear increase because of significantly weaker shoulders marked with A and B. The intensity at the principal edge-maximum at (C) was clearly reduced only in the oxygen evolving active state (at +1.62 V). Samples were freeze-quenched under potential control using liquid N_2_ after 15 min at the given potential in 0.1 M KP_i_ and stored in liquid N_2_ until XAS measurements were conducted.

To determine the local atomic structure and oxidation state of the Co ions in Co_3_O_4_ under electrochemical reaction conditions and, especially, the Co coordination in the X-ray amorphous CoO_*x*_ present during OER, we performed X-ray absorption spectroscopy at the Co *K*-edge for Co_3_O_4_ films, which were deposited on GC and freeze-quenched at selected electrode potentials. In a previous work^27^, we have verified that the protocol of this experiment facilitates the collection of high-quality, extended-range EXAFS data that remain unaffected by radiation-induced modifications of the solvent-exposed catalyst material. Figure 5 shows the X-ray absorption near-edge structure (XANES) spectra recorded at the Co *K*-edge of Co_3_O_4_ films at selected catalyst states. The energy of the Co *K*-edge of the as-prepared catalyst agreed well with a mean Co oxidation state between +2 and +3, as expected for Co_3_O_4_. With increasing electrode potential, the Co absorption edge shifted slightly to higher energies, which is indicative for a higher mean oxidation state of the Co ions^51^.

During OER, the XANES profile exhibited an almost linear increase due to significantly weaker shoulders at about 7,718 (A) and 7,723 eV (B); moreover, a clearly reduced intensity at the principal edge-maximum at about 7,729 eV (C) was observed only in the OER-active state (at +1.62 V). These spectroscopic differences indicate significant structural differences in the local atomic and electronic structure of the amorphous CoO_*x*_ shell formed during catalysis (at +1.62 V) as compared with the crystalline Co_3_O_4_ structure prevalent before and after catalysis. However, as expected from the *in situ* GIXRD results, the majority of Co ions remained in their initial state within the crystalline Co_3_O_4_.

**Figure 6:**
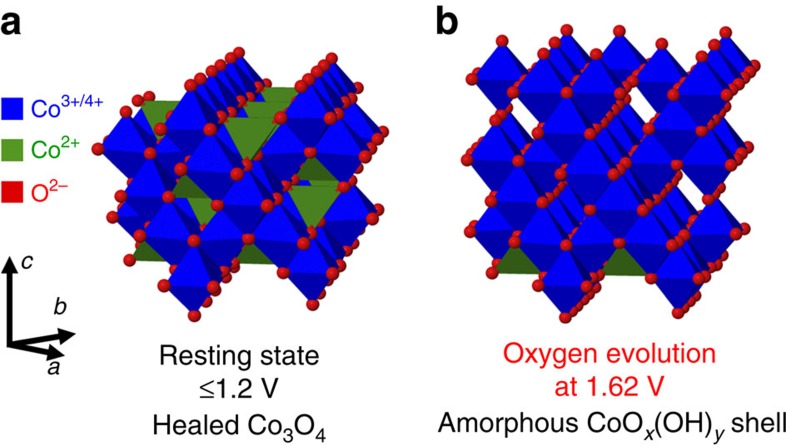
Possible near-surface structures on crystalline Co_3_O_4_ core under electrochemical conditions. At potentials below Co redox features (**a**), Co_3_O_4_ is in a healed state at which defects in the near-surface are oxidized. At elevated O_2_ evolution (**b**), the CoO_*x*_(OH)_*y*_ grows into the crystalline Co_3_O_4_ core leading to a reversible amorphization of a sub-nanometre shell. This amorphous CoO_*x*_(OH)_*y*_ shell consists of di-μ-oxo-bridged Co^3+/4+^ ions with arbitrary site occupancy in the ideal cubic close-packed O^2−^ lattice. Hydrogen atoms and phosphates are not shown.

The local atomic structure of the absorbing Co ions could be assessed qualitatively from the Fourier-transformed EXAFS and quantitatively by EXAFS simulations (Supplementary Table 2). In Fig. 3b, three major peaks representing absorber-backscatterer distances are visible for the Co_3_O_4_ films in all catalyst states. The first peak corresponds to the convolution of two Co–O distances stemming from tetrahedral and octahedral Co coordination (mean distance of ∼1.91 Å). The second and third peak is consistent with the Co–Co distances for pairs of Co ions connected via di-μ-oxo (∼2.85 Å) and mono-μ-oxo (∼3.36 Å) bridges, respectively. Di-μ-oxo bridges represent edge-sharing Co octahedra, whereas the mono-μ-oxo bridges reflect corner-sharing Co octa- and tetrahedra (cf. Fig. 6a). Comparing the magnitude of the second and third FT-peak, significant changes in the Co coordination during oxygen evolution could be identified, in particular the shift towards more di-μ-oxo-bridged, octahedrally coordinated Co ions in the OER-active state (at +1.62 V).

Figure 4b shows the energy shift of the Co *K*-edge compared with the as-prepared state for various catalyst states. The edge position was identical at +1.0 and +1.2 V but was shifted by +0.1 eV compared with the as-prepared state. At +1.55 V, the edge position was +0.3 eV higher and the determined shift was maximal at high OER activity. After OER, the edge position of the Co_3_O_4_ agreed well with the as-prepared state. Thus, the evolution of the Co oxidation state is a reversible process and the mean oxidation state for electrode potentials at +1.0 V and above is higher than under dry conditions.

Simulations of k^3^-weighted EXAFS spectra were conducted to determine the Co coordination in Co_3_O_4_ under electrochemical reaction conditions and especially of the X-ray amorphous CoO_*x*_ present during OER (Supplementary Fig. 14 and Supplementary Table 2). Figure 4c shows the relative changes in the coordination numbers assignable either to di- or mono-μ-oxo bridges at selected catalyst states. In agreement with the evolution of the structural coherence length, the number of di- and mono-μ-oxo bridges remained constant within the experimental error between the as-prepared state and +1.55 V. The reversible amorphization at elevated oxygen evolution rate, however, was accompanied by a change from mono- towards di-μ-oxo-bridged Co ions; the *ex situ* state after OER resembled the as-prepared state in terms of μ-oxo Co linking.

In summary, the X-ray absorption data suggest that in the OER-active state with elevated levels of oxygen evolution (at +1.62 V), tetrahedrally coordinated, mono-μ-oxo-bridged Co^2+^ ions are reversibly converted into octahedrally coordinated, di-μ-O(H)-bridged Co^3+/4+^ ions. Analysis of the X-ray diffraction data reveals a simultaneous coherence-length reduction by 5–10 Å, suggesting a smaller crystalline Co_3_O_4_ core in the OER active state. Both phenomena, the increased amount of di-μ-O(H)-bridged Co^3+/4+^ octahedra and the reduced size of the crystalline Co_3_O_4_ core, are reversed upon returning to the resting state of the catalyst. These observations are consistently explainable by a reversible transformation of part of the Co_3_O_4_ core into a CoO_*x*_(OH)_*y*_ surface shell, as discussed in more detail further below.

### Surface chemistry and electronic structure

For further insights in the elemental composition, atomic and electronic structure of the near-surface of the Co_3_O_4_ crystallites at the electrode/electrolyte interface before and after OER, we conducted synchrotron-based X-ray photoemission (XPS) and XANES spectroscopy at Co *L*- and O *K*-edge on the Co_3_O_4_ films. Supplementary Fig. 17a,b shows Co 2p XPS and the Co *L*_*3*_ XANES spectra of Co_3_O_4_ films, respectively. Both spectra support the notion of a Co oxide containing mainly Co_3_O_4_ in the near-surface of the crystallites^52^. We note that because of the more pronounced fine structure at the low-energy side of the Co L_3_ absorption peak, the presence of a minor fraction of Co^2+^ O_h_ in the near-surface is possible (cf. Supplementary Fig. 19). The O 1 s XPS showed that the near-surface of the as-prepared state incorporated protonated oxygen atoms in addition to lattice oxygen (cf. Supplementary Figs 17c and 20). The strong pre-edge feature of the O *K*-edge XANES spectrum revealed significant hybridization of Co 3d and O 2p orbitals (cf. Supplementary Fig. 17d)^53^.

After OER, the near-surface of the Co_3_O_4_ crystallites was largely unchanged; XPS and XANES spectra showed almost identical profiles when compared with the as-prepared catalyst material. Minor differences can be accounted for by adsorption of phosphate ions during operation of the catalyst film in the phosphate-containing electrolyte (cf. Supplementary Fig. 18). We conclude that the identified reversibility of the structural transformation observed by X-ray diffraction and X-ray absorption spectroscopy at the Co *K*-edge is fully supported by our surface-sensitive spectroscopic data (see Supplementary Note 1 for details).

## Discussion

We have investigated the structural evolution of crystalline Co_3_O_4_ films under electrochemical potential control and during OER in neutral, phosphate-containing electrolyte in detail using *in situ* GIXRD and quasi-*in situ* XAS. For the first time, we uncover a reversible decrease in structural coherence length at electrochemical potentials facilitating elevated oxygen evolution, which is coupled to Co oxidation and a change in Co coordination from tetrahedral towards octahedral symmetry. In line with the reversibility, composition and electronic structure of the Co_3_O_4_ in the bulk volume and in the crystallites near-surface zone remained nearly identical when comparing the catalyst material before and after OER.

To explain this reversible process, we propose that the changes in Co coordination at elevated oxygen evolution rate are caused by formation of a three-dimensional (3D) cross-linked CoO_*x*_(OH)_*y*_ structure. Figure 6 sketches the structural transformation of the near-surface structure of the crystallites between the resting state (below the Co redox features) and the catalytically active state (at +1.62 V). At potentials below the Co redox features (at +1.2 V), the Co_3_O_4_ is in a healed state with slightly higher mean oxidation state than in the dry state. Reduced Co sites caused by defects in the near-surface of the as-prepared state are oxidized to Co_3_O_4_. At potentials above the Co redox features at the onset of OER, Co_3_O_4_ is partially oxidized to a CoO_*x*_(OH)_*y*_ without changing crystallinity and local atomic coordination. Thus, the oxidation is presumably limited to the outermost surface of the crystallites. The formation of a layered CoOOH in the near-surface can be excluded because it would diminish the structural isotropy to the Co_3_O_4_ core and thus, lead to a lower structural coherence length already at +1.55 V. Bulk oxidation of Co_3_O_4_ appears to be kinetically hindered and oxidation is thus limited to the surface of the crystallites at +1.55 V. Therefore, we conclude that the onset of the electrocatalytic oxygen evolution occurs on crystalline Co_3_O_4_, which is oxidized to CoO_*x*_(OH)_*y*_ at the outermost surface (initial active surface). We propose this CoO_*x*_(OH)_*y*_ represents the reaction zone of the OER. At elevated oxygen evolution rate, the crystallinity decreases and the local atomic structure of Co ions in the near-surface changes. We explain this amorphization with the growth of the CoO_*x*_(OH)_*y*_ reaction zone from the surface into the crystalline Co_3_O_4_ core.

With increasing electrode potential and oxygen evolution rate, the Co^2+^ T_d_ ions of the crystalline Co_3_O_4_ in the near-surface get oxidized to +3 and change their coordination from tetrahedral to octahedral. Co^4+^ ions can be isostructurally incorporated in the CoO_*x*_(OH)_*y*_ via deprotonation of di-μ-OH bridges^27^. To undergo this change in coordination, Co ions have to move inside the ccp O^2−^ lattice to vacant O_h_ sites of the reaction zone of the CoO_*x*_(OH)_*y*_. Site occupancy of the Co ions can be arbitrary and dynamic under the constraints of an appropriate O/Co ratio and sustained prevalence of the di-μ-oxo Co-bridging motif. As a consequence of the unified Co coordination and thus, unified Co–O and Co–Co distances in the CoO_*x*_(OH)_*y*_, a slight rearrangement of the O^2−^ lattice towards an ideal ccp arrangement occurs. These structural changes diminish the structural isotropy to the crystalline Co_3_O_4_ core and lead to the identified amorphization. After oxygen evolution, the reaction zone transforms back to the thermodynamically favoured spinel structure.

The origin of the structural transformation of the near-surface described above can furthermore be described by participation of lattice oxygen in the OER mechanism, in which the temporary presence of O vacancies in the CoO_*x*_(OH)_*y*_ also induce structural disorder in the reaction volume^45^,^54^,^55^. In this scheme, before OER the CoO_*x*_(OH)_*y*_ surface gets oxidized but is thermodynamically frustrated when oxidation equivalents are accumulated at higher electrode potentials. Above a critical concentration of oxidation equivalents OER starts possibly under participation of lattice oxygen. The formation of oxygen vacancies then initiates structural relaxation, which leads to the described change in Co coordination and amorphization in the reaction zone. The short life-time of the O vacancies precludes the reduced Co sites from identification using quasi-*in situ* XANES (cf. Figs 4 and 5).

3D cross-linked Co oxides form also from Co_3_O_4_ during Li insertion, which underlines the flexibility of Co ions within the ccp O^2−^ lattice under electrochemical conditions^56^,^57^. Under oxygen evolving conditions, octahedral Co coordination and a mean oxidation state of higher than +3 appears to be a general structural motif as it has been also found for the CoCat^27^,^28^ and layered CoO_*x*_(OH)_*y*_ (refs 43, 58). The 3D cross-linked CoO_*x*_(OH)_*y*_ appears also structurally similar to the rutile-type oxides such as RuO_2_, IrO_2_ and β-MnO_2_ (refs 11, 12, 13, 17, 31). Both structures exhibit octahedral chains of di-μ-oxo-bridged metal ions and tunnels in the oxide lattice.

We note that the uncovered amorphization of the near-surface is likely to have significant, beneficial influence on the electrocatalysis. An amorphous metal oxide provides an enhanced degree of structural flexibility, a higher number of special coordination sites possibly under participation of several metal centres and enables elevated accumulation of oxidation equivalents in the reaction zone. *Vice versa*, perfect crystalline order seems to prevent efficient oxygen evolution. The reported OER activity of several crystalline oxide materials may relate directly to reversible surface amorphization ‘templated' by the crystalline core of the material^13^,^59^,^60^. Thus, a reversible formation of an amorphous termination layer on a stable crystalline core points towards a unified common oxygen-evolving state of Co oxide (but also other) electrocatalysts—independent of its initial degree of order. This may provide a new approach towards a unified understanding of heterogeneous water oxidation catalysis. Furthermore, we propose that the design of more active and stable electrocatalysts is possible by the preparation of core-shell nanostructures in which near-surface amorphization (under electrochemical conditions) is enhanced and simultaneously the crystalline metal oxide core stabilizes the electrocatalyst from elevated metal ion dissolution.

In conclusion, using *in situ* X-ray diffraction and quasi-*in situ* X-ray absorption spectroscopy at the Co *K*-edge as well as XPS and XANES at Co *L*- and O *K*-edge before and after OER in neutral electrolyte, we have identified the existence of a reversible structural transformation of Co_3_O_4_, which accompanies elevated catalytic oxygen evolution. Below the Co redox features, the bulk structure of the Co_3_O_4_ is in a stable rest state with larger size of the crystalline core compared with the dry state. At the onset of the OER catalysis, the Co oxidation state increases within the pseudo-ccp O^2−^ lattice of the reaction zone under retention of the Co coordination. As a result of this, the CoO_*x*_(OH)_*y*_ structure remains limited to the surface of the crystallites. However, this catalytic reaction zone grows into the crystalline Co_3_O_4_ core at more anodic electrode potentials and enhanced oxygen evolution. This is linked to the (reversible) amorphization of a sub-nanometre shell. The amorphization is accompanied by a higher Co oxidation state and a partial change in Co coordination from tetrahedral to octahedral and can be explained by arbitrary site occupancy of Co^3+/4+^ ions within a slightly rearranged ccp O^2−^ lattice. On the reverse cathodic electrode potential scan, more negative of the active catalytic regime, the amorphous shell crystallizes back to its initial state.

Structurally, we propose that the formation of an CoO_*x*_(OH)_*y*_ with di-μ-oxo-bridged Co^3+/4+^ O_h_ ions at the surface is *sine qua non* for oxygen evolution activity on Co_3_O_4_. At elevated oxygen evolution, the amorphous 3D cross-linked CoO_*x*_(OH)_*y*_ offers the structural flexibility that enables and enhances catalytic activity. The crystalline Co_3_O_4_ combines the advantages of a controlled and stable crystalline material in resting state with the required structural flexibility facilitated by a non-crystalline oxide under OER conditions. The notion of and first evidence for a reversible amorphization mechanism, in which electrocatalysts move reversibly between crystalline and amorphous regimes as a response to externally applied electrode potentials, is a new and potentially very important design concept for catalysts.

## Methods

### Synthesis and bulk characterization

Co oxide films were deposited on GC (HTW) plates using spin coating. Before the deposition, the substrates were polished until a mirror-like finish was achieved and cleaned stepwise in an ultrasonic bath using de-ionized water and acetone. The Ti cylinders were treated in HNO_3_/H_2_O solution for 2 h at 150 °C after polishing.

The spin coating solution consisted of 0.25 M Cobalt(II) 2,4-pentanedionate (AlfaAesar) in 15 vol.% acetic acid (Sigma-Aldrich, 99.7%), 18 vol.% de-ionized water (≥18 MΩ) and 67 vol.% ethanol (abs., AnalaR Normapur). The precursor was dissolved using sonification for 10 min. The spin coating was conducted at 2,000 r.p.m. for the GC plates and 5,000 r.p.m. for Ti cylinders. In total three and five layers were deposited on the GC and Ti substrates, respectively. After each layer deposition, the samples were calcined in air for 10 min at 300 °C to decompose the Co precursor. Finally, the samples were calcined for 15 min at 400 °C in air to achieve a crystalline Co oxide.

The Cobalt loading has been determined for a one layer deposition on a GC substrate by ICP-OES (Varian 715-ES) to be 320 nmol_Co_ cm^−2^. Therefore, the samples were immersed in a HCl/HNO_3_ mixture overnight and the solution diluted using de-ionized water. Scanning electron microscopy images were acquired in secondary electron mode with a JEOL 7401F field emission scanning electron microscopy operated at 10 kV. Transmission electron micrographs were recorded using a FEI Tecnai G^2^ 20S-TWIN instrument operated at 200 kV. Metal oxide films were scratched off the substrate using scalpel and transferred onto a lacy carbon-coated copper grid.

### Electrochemical measurements

Electrocatalysts were tested using a three electrode rotating disk electrode setup in a custom-made glass cell using a PINE rotator with a custom-made sample holder and a Biologic SP-200 potentiostat. Platinum gauze acted as a counter electrode and a mercury/mercury sulphate electrode connected via a Haber–Luggin capillary was used as a reference electrode. The reference electrode was freshly calibrated versus a Pt/H_2_ electrode. Electrochemical experiments were conducted in 0.1 M phosphate buffer (KPi) at pH 7 prepared by mixing 0.1 M K_2_HPO_4_ (99.99%, Merck Suprapur) and 0.1 M KH_2_PO_4_ (99.995%, Merck Suprapur) aqueous solutions until the desired pH was obtained. Before electrochemical measurements, N_2_ was bubbled through the electrolyte for at least 15 min and was continuously bubbled during experiments. The working electrode was rotated at 400 r.p.m. Unless differently stated, all electrode potentials had been corrected for Ohmic losses using electrochemical impedance spectroscopy and are referred to the reversible hydrogen electrode (RHE). Impedance spectra were fitted using an equivalent electrical circuit consisting of a serial connection of an Ohmic resistance, resistor–capacitor circuit (RC) circuit and a constant phase element. A specific capacitance of 35 μF cm^−2^ was used to calculate electrochemical surface area from the determined capacitance^14^. The electrochemical protocol consisted of a dynamic OER activity test, a series of cyclic voltammograms with elevated sweep rates, an additional dynamic OER activity test, a quasi-stationary potential step OER activity test and again a dynamic OER activity test followed by a series of cyclic voltammograms with elevated sweep rates. The dynamic OER activity test consisted of a potentiostatic impedance spectroscopy recorded at 1.0 V in the frequency range between 50 kHz and 1 Hz using a modulation amplitude of 20 mV and one CV between 1.0 V and E(i=5 mA cm^−2^) with a sweep rate of 6 mV s^−1^. The first dynamic activity test consisted of two CVs at 6 mV s^−1^. The series of cyclic voltammograms consisted of 20 cycles with sweep rate of 500 mV s^−1^ followed by each three cycles of 200, 100, 50 and 20 mV s^−1^. For the quasi-stationary OER activity test, the electrode potential was increased from +1.50 to +1.72 V using steps of 20 mV, or vice versa. Each potential step was hold for ∼4 min and an impedance spectrum was recorded.

### DEMS measurements

To investigate the reaction products, DEMS was performed. The DEMS apparatus consisted of a home-made dual thin-layer electrochemical flow cell based on a design reported elsewhere^61^. The flow cell was connected via separation polytetrafluoroethylene (PTFE) membrane to a Prisma quadrupole mass spectrometer (QMS 200, Pfeiffer Vacuum) equipped with two turbomolecular pumps HiPace 80 operating the MS chamber at 10^−6^ mbar. N_2_-saturated 0.1 M KPi at pH 7 acted as electrolyte and the electrolyte flow was adjusted to 5 μl s^−1^. The reference electrode was a reversible hydrogen electrode and a Pt wire was the counter electrode. Cyclic voltammetry measurements were conducted in the OER regime between 1.1 and 1.675 V versus reversible hydrogen electrode using a sweep rate of 6 mV s^−1^. Simultaneously, the ion current for O_2_ (*m*/*z*=32) and CO_2_ (*m*/*z*=44) was recorded. All electrode potentials were corrected for Ohmic losses.

### *In situ* GIXRD measurements

Investigation of crystal structure under electrochemical reaction conditions have been conducted at the beamline of the Max-Planck-Institute for Solid State Research at the Angströmquelle Karlsruhe (ANKA)^62^. An home-made *in situ* electrochemical cell for X-ray studies based on the thin-layer concept was used^63^. The electrolyte was constantly circulated using a peristaltic pump and degassed with N_2_ during *in situ* GIXRD studies. A Ag/AgCl (3 M KCl, World Precision Instruments, freshly calibrated versus a Pt/H_2_ electrode) and a Pt wire acted as reference electrode and counter electrode, respectively. The working electrode was immersed at +1.0 V and then the electrode potential increased to the desired potential with 6 mV s^−1^. Before X-ray characterization, the electrode potential was hold for at least 10 min to ensure stationary conditions. The OER potential was +1.62 V, which was determined from current density of 0.5 mA cm^−2^ during the anodic potential scan. After OER, the electrode potential was decreased to +1.0 V and the electrode removed under potential control. Peak broadening was determined by fitting the reflections of Co_3_O_4_(511), Ti(110) and Co_3_O_4_(440) using pseudo-Voigt profiles. The structural coherence length was calculated using the Scherrer equation from the integral breadth of the fitted Co_3_O_4_ profiles using a shape factor of 0.89. Errors of the structural coherence length were calculated from the estimated s.d. of the integral breadths.

### X-ray absorption measurements

Co *K*-edge experiments were carried out at the BESSY synchrotron radiation source operated by the Helmholtz–Zentrum Berlin. The measurements were conducted at the KMC-1 bending-magnet beamline at 20 K in a cryostat (Oxford-Danfysik) with a liquid-helium flow system. Further details are given in ref. 10. Electrochemical conditioning was conducted in analogy to the *in situ* GIXRD experiment. After 15 min at the desired electrode potential, the samples were freeze-quenched using liquid N_2_ under potential control and stored in liquid N_2_ until XAS measurements were conducted. Further information regarding data analysis is given in the caption of Supplementary Table 2.

### XPS and XANES at Co *L*- and O *K*-edge measurements

Co, O and P core electron emission as well as Co *L*- and O *K*-edge absorption spectra were collected at the ISISS beamline^64^ of the synchrotron radiation facility BESSY of the Helmholtz–Zentrum Berlin. The kinetic energy of the photoelectrons recorded during XPS was 550 eV. The XANES spectra were recorded in the total electron yield mode. Electrochemical conditioning was conducted at +1.62 V for 15 min in 0.1 M KP_i_ at pH 7. Samples were extensively washed using de-ionized water directly after removal from the electrolyte and dried in N_2_ flow.

## Additional information

**How to cite this article:** Bergmann, A. *et al*. Reversible amorphization and the catalytically active state of crystalline Co_3_O_4_ during oxygen evolution. *Nat. Commun.* 6:8625 doi: 10.1038/ncomms9625 (2015).

## Supplementary Material

Supplementary Information Supplementary Figures 1-20, Supplementary Table 1-2, Supplementary Note 1 and Supplementary References
