# Supplementary material for: Reversible amorphization and the catalytically active state of crystalline Co3O4 during oxygen evolution
Source: Nat Commun. 2015 Oct 12;6:8625. doi: 10.1038/ncomms9625 (PMC4633955; doi:10.1038/ncomms9625)
Supplement: Supplementary Information — Supplementary Figures 1-20, Supplementary Table 1-2, Supplementary Note 1 and Supplementary References [file ncomms9625-s1.pdf]

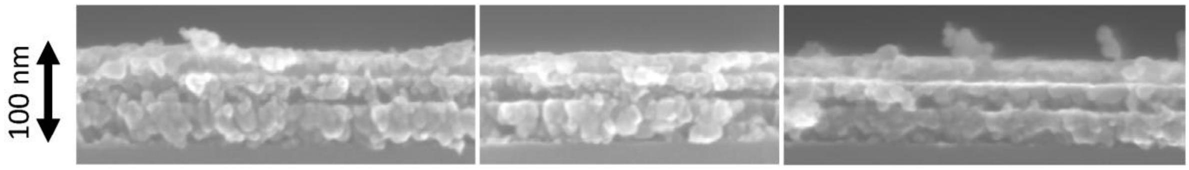

Supplementary Fig. 1: Cross-section scanning electron micrographs of as-prepared Co_3O_4 films deposited on Si (100) wafer. Film thickness is 85 ± 8 nm. The Co loading of the same sample is 294 ± 14 $nmol_{Co}\cdot cm^{-2}$ as determined from ICP-OES. The porosity of the Co_3O_4 films is 0.46 by assuming ideal Co_3O_4 with a molecular weight of 240.7956 $g\cdot mol^{-1}$ and a density ρ of 6.07 $g\cdot cm^{-3}$.

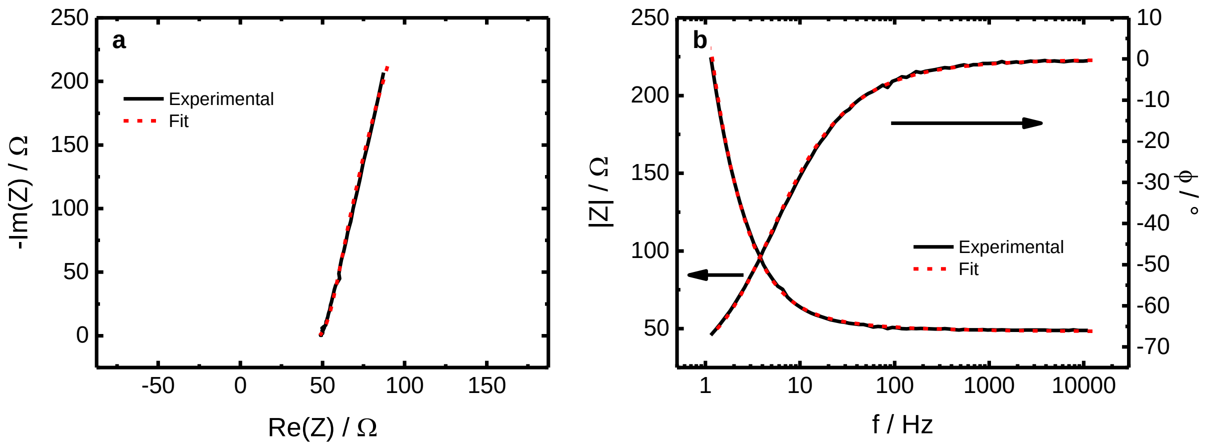

Supplementary Fig. 2: Potentiostatic electrochemical impedance spectra of Co_3O_4 films deposited on glassy carbon recorded at 1.0 V. Impedance spectra were fitted using an equivalent electrical circuit consisting of a serial connection of an Ohmic resistance, RC circuit and a constant phase element. A specific capacitance of 35 μF cm^{-2} was used to calculate ECSA from the determined capacitance.[1]

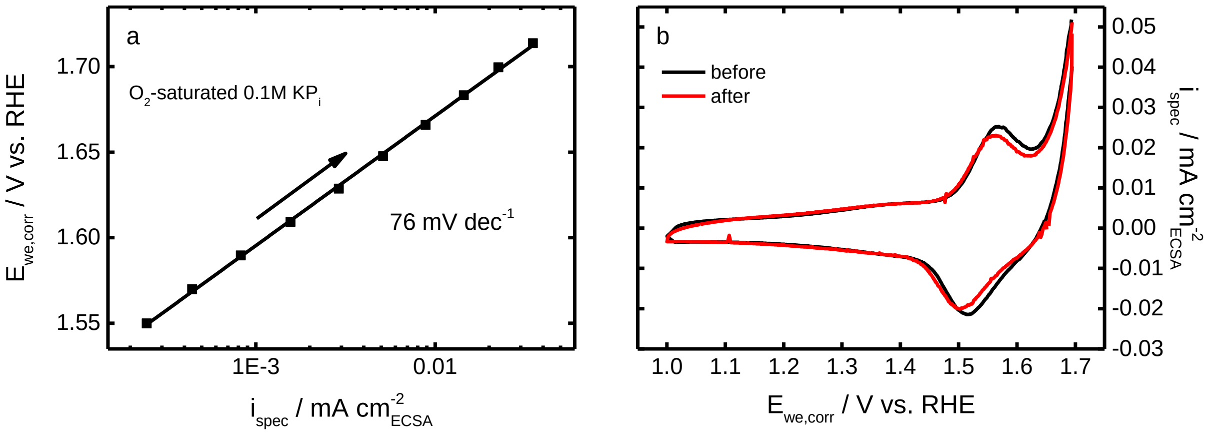

Supplementary Fig. 3: Quasi-stationary anodic potential step experiment of Co_3O_4 films performed in O_2-saturated 0.1M KP_i (a) and cyclic voltammograms (b) recorded at 50 mV/s in N_2-saturated 0.1M KPi at pH 7 before (black) and after (red) the quasi-stationary cathodic potential step experiment performed in O_2-saturated electrolyte. The Tafel slope is 76 mV/dec while the exchange current density is $1.52 \cdot 10^{-8}$ $mA \cdot cm^{-2}{}_{ECSA}$. Current was normalized using the initial ECSA as determined by PEIS. Electrode potentials were corrected for Ohmic losses using PEIS.

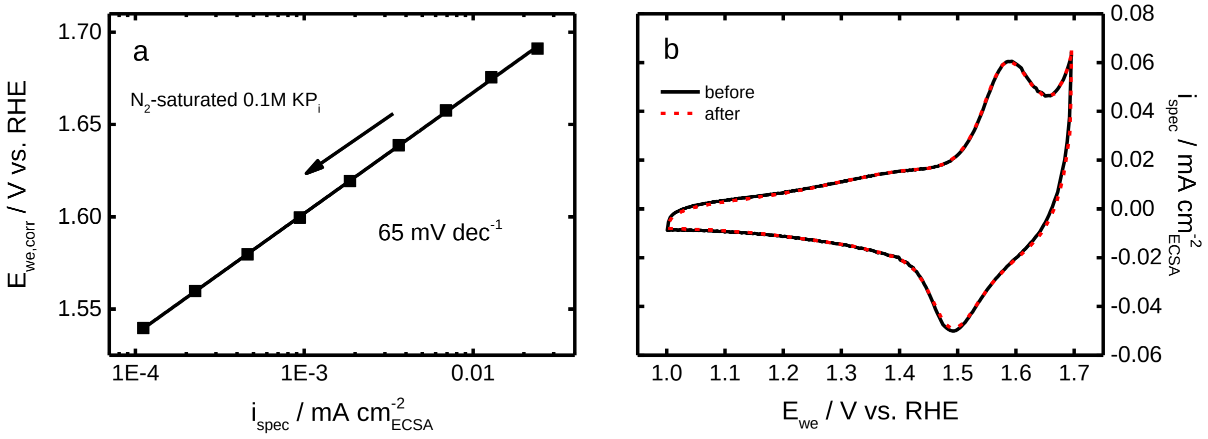

Supplementary Fig. 4: Quasi-stationary cathodic potential step experiment of Co_3O_4 films performed in N_2-saturated 0.1M KP_i (a) and cyclic voltammograms (b) recorded at 100 mV/s in N_2-saturated 0.1M KPi at pH 7 before (black) and after (red, dashed) the quasi-stationary cathodic potential step experiment. The Tafel slope is 65 mV dec^{-1} while the exchange current density is $2.09 \cdot 10^{-9}$ mA $cm^{-2}{}_{ECSA}$. Current was normalized using the initial ECSA as determined by PEIS. Electrode potentials were corrected for Ohmic losses using PEIS.

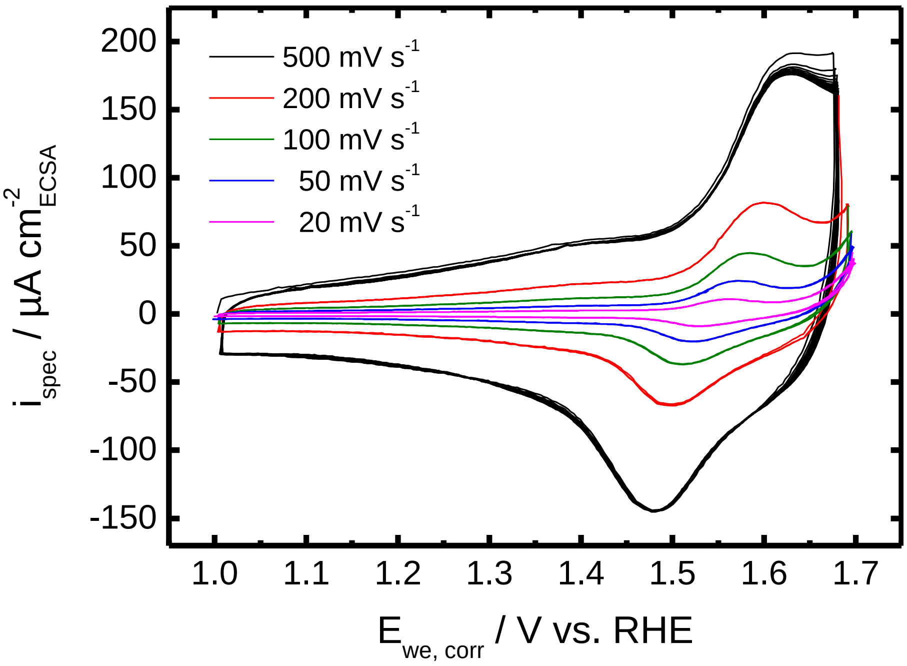

Supplementary Fig. 5: Series of cyclic voltammograms of Co_3O_4 thin films recorded at the given sweep rates in N_2-sat. 0.1M KPi at pH 7. The cyclic voltammograms were recorded after two initial CVs recorded with a sweep rate of 6 mV s^{-1} between 1.0 and 1.76 V as shown in Supplementary Fig. 6. Current was normalized using the initial ECSA as determined by PEIS. Electrode potentials were corrected for Ohmic losses using PEIS.

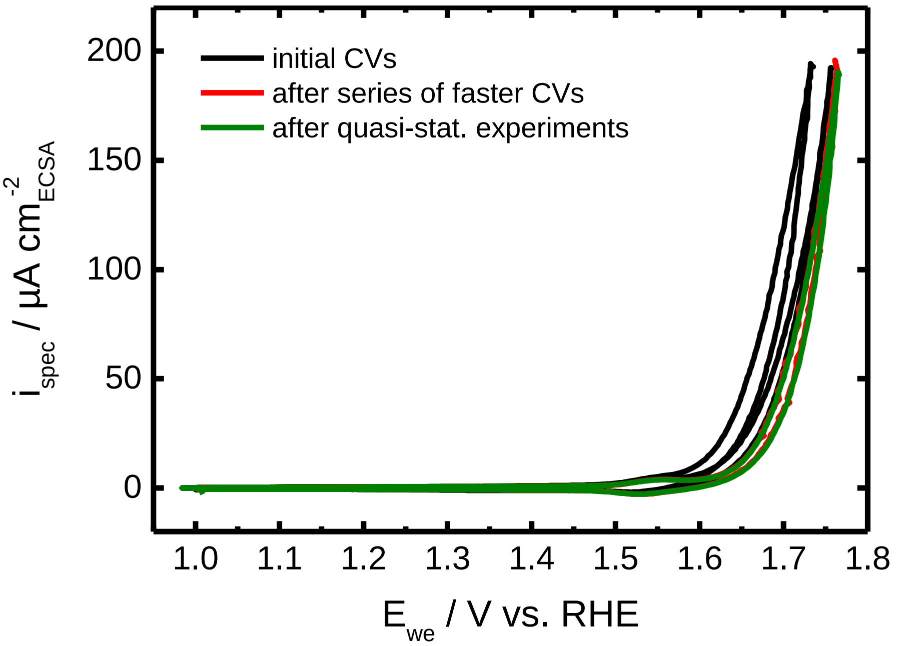

Supplementary Fig. 6: Cyclic voltammograms of Co_3O_4 thin films recorded at 6 mV s^{-1} in N_2-saturated 0.1M KPi at pH 7 at different stages of the electrochemical characterization protocol. The initial irreversible loss of current can be explained by the electrochemical oxidation of organic, carbonaceous residues adsorbed on the Co_3O_4 surface. Current was normalized using the initial ECSA as determined by PEIS prior to OER. Electrode potentials were corrected for Ohmic losses using PEIS.

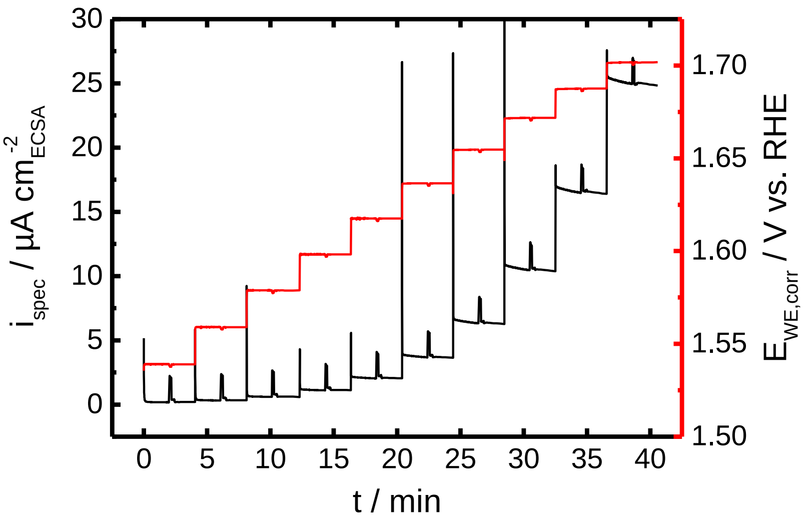

Supplementary Fig. 7: Current and potential vs. time profile of quasi-stationary potential step experiments of Co_3O_4 films recorded in anodic direction in N_2-saturated 0.1M KPi at pH 7. Each electrode potential was hold for ~4 min and an electrochemical impedance spectra (current spikes) was recorded at each potential step. Current was normalized using the initial ECSA as determined by PEIS. Electrode potentials were corrected for Ohmic losses using PEIS.

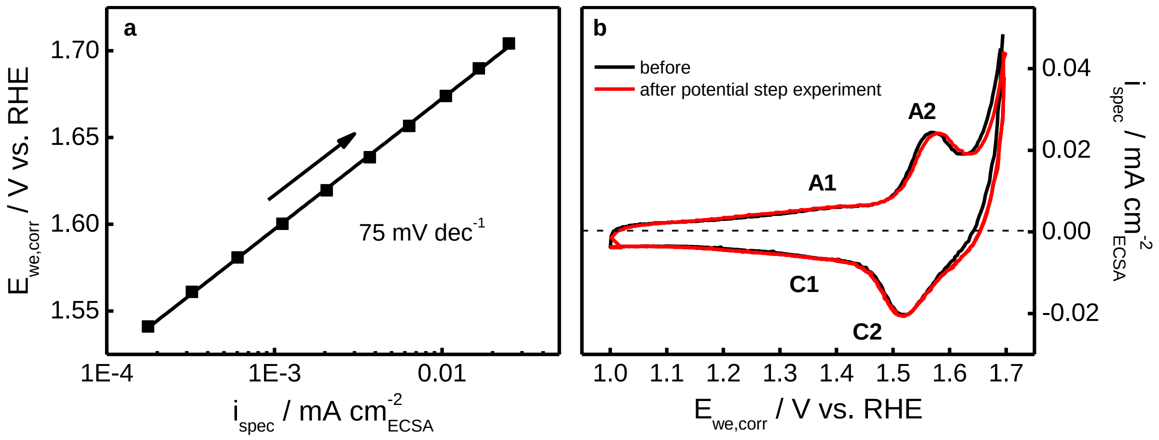

Supplementary Fig. 8: Tafel plot (a) and cyclic voltammograms (b) of Co_3O_4 thin films recorded at 50 mV s^{-1} in N_2-saturated 0.1M KPi at pH 7 before and after the quasi-stationary anodic potential step experiment. Current was normalized using the initial ECSA as determined by PEIS. Electrode potentials were corrected for Ohmic losses using PEIS.

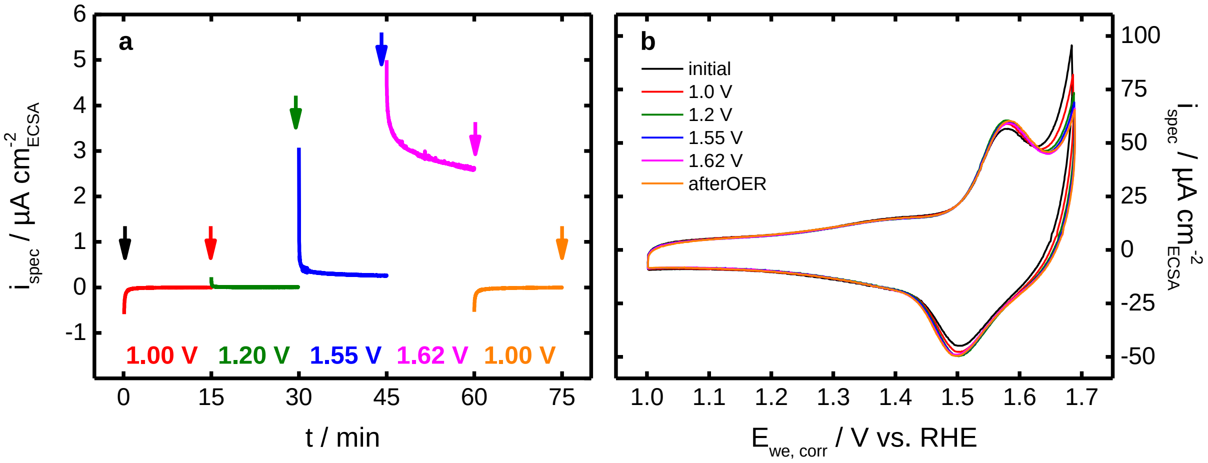

Supplementary Fig. 9: Current-time profile (a) of potential step experiments at the electrode potentials selected for in situ characterization and cyclic voltammograms (b) recorded with 100 mV s^{-1} after 15 min at the corresponding electrode potentials. The 10th cycle is shown. N_2-saturated 0.1M KPi at pH 7 acted as electrolyte. Current was normalized using the initial ECSA as determined by PEIS. Electrode potentials were corrected for Ohmic losses using PEIS.

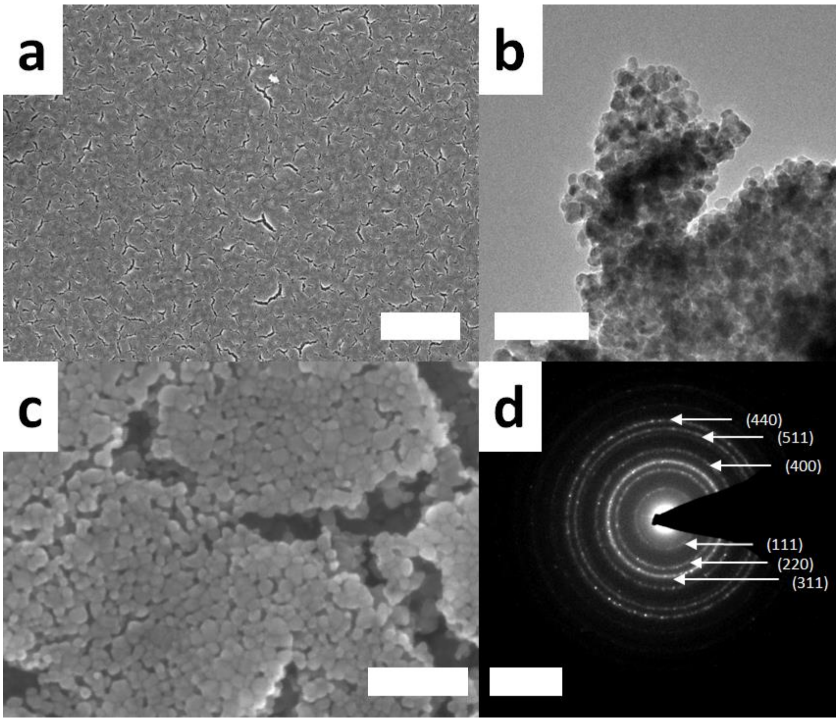

Supplementary Fig. 10: Scanning electron micrographs in low (A) and high magnification (C), transmission electron micrograph (B) and selected area electron diffraction (SAED) pattern (D) of Co_3O_4 films after OER. Diffraction rings of Co_3O_4 are indexed in the SAED pattern. Micrographs and diffraction pattern were recorded after 15 min of OER at 1.62 V in 0.1M KP_i. After 15 min of OER at 1.62 V in 0.1M KPi approximately 1% of the initial Co loading was dissolved in the electrolyte. The scale bars represent 2 µm, 50 nm, 100 nm, and 5 nm^{-1} in panel a, b, c, and d, respectively.

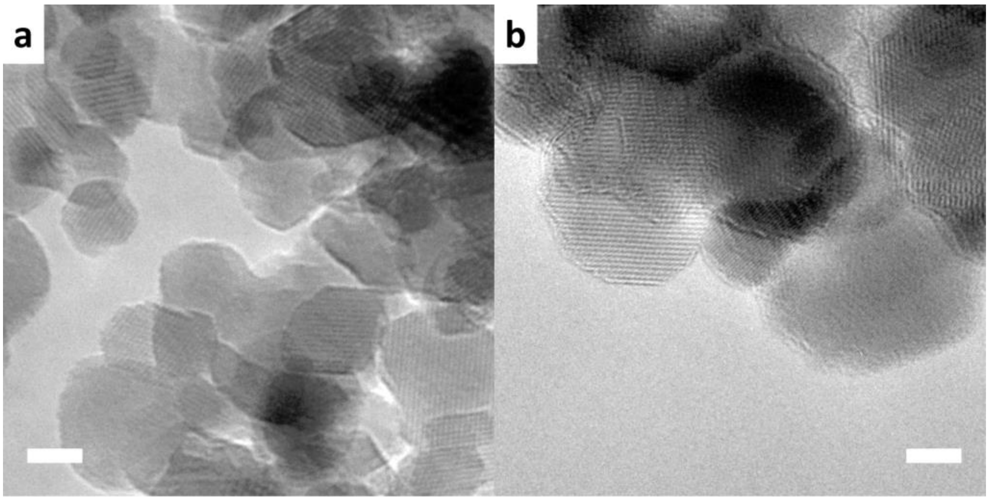

Supplementary Fig. 11: Transmission electron micrograph of Co_3O_4 films (a) in the as-prepared state and (b) after 15 min at 1.62 V in 0.1M KPi at pH 7 without electrochemical reduction after electrocatalysis in the left and right panel, respectively. Transmission electron micrographs recorded after OER show no presence of a restructuring in the near-surface due to cationic redistribution but some defects in the outermost surface layer of the Co_3O_4 crystallites. The scale bars represent 5 nm.

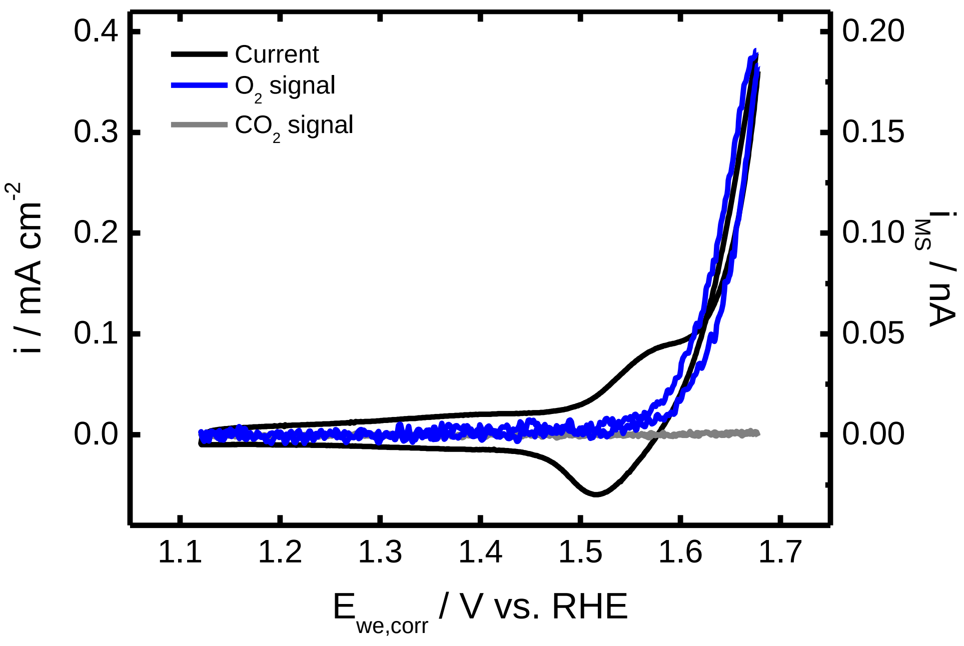

Supplementary Fig. 12: Cyclic voltammogram (black) of Co_3O_4 film deposited on glassy carbon recorded at 6 mV s^{-1} in 0.1M KPi at pH 7 as well as the corresponding signals of m/z=32 (blue) and m/z=44 (grey) corresponding to the signals of O_2 and CO_2, respectively. The electrode potential was corrected for Ohmic losses using PEIS and current was normalized using geometrical sample area.

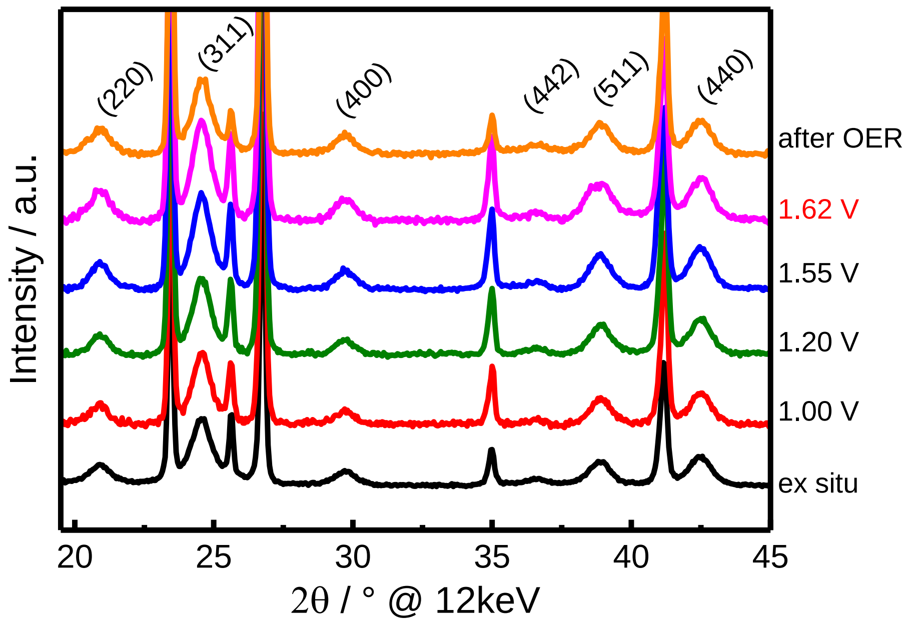

Supplementary Fig. 13: *in situ* X-ray diffraction patterns of Co_3O_4 catalyst films. The diffraction patterns were recorded using grazing-incident excitation at α=0.3° and 12 keV. The electrode potential was increased stepwise from 1.0 V to 1.62 V vs. RHE, the latter representing the catalytically-active state, in 0.1 M KPi at pH 7. The state after OER is a dry state for which the electrode was removed from electrolyte at 1.0 V rinsed with de-ionized water and dried in N_2 flow. The Miller indices of selected Co_3O_4 reflections are indicated. Diffraction pattern were background corrected for better visualization.

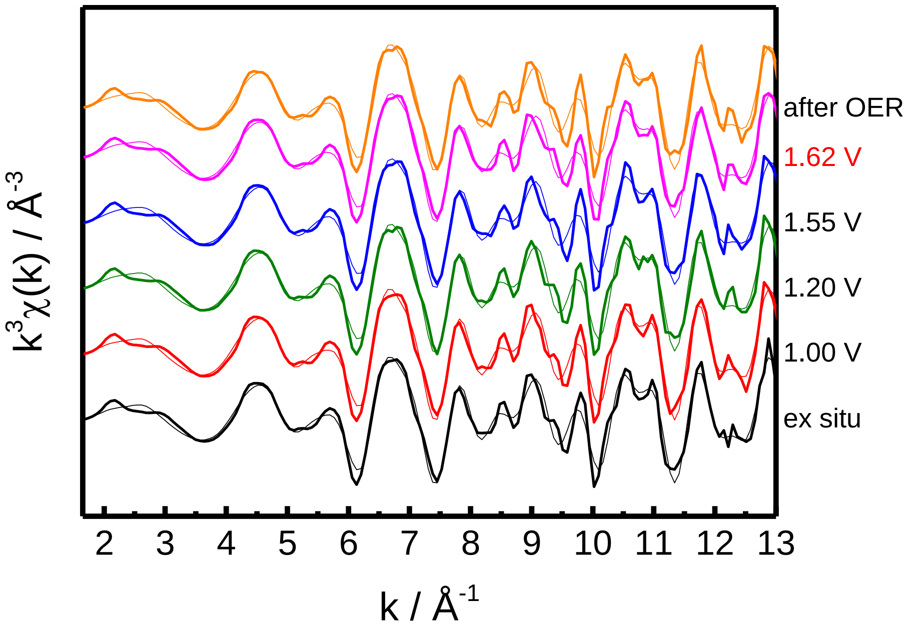

Supplementary Fig. 14: quasi-*in situ* k^3-weighted EXAFS spectra of Co_3O_4 thin films recorded at Co *K*-edge as a function of catalyst state. Experimental and simulated spectra are shown in bold and thin, respectively. Samples were freeze-quenched using liquid N_2 under electrochemical potential control after electrochemical conditioning for 15 min at the given potential in 0.1M KPi at pH 7.

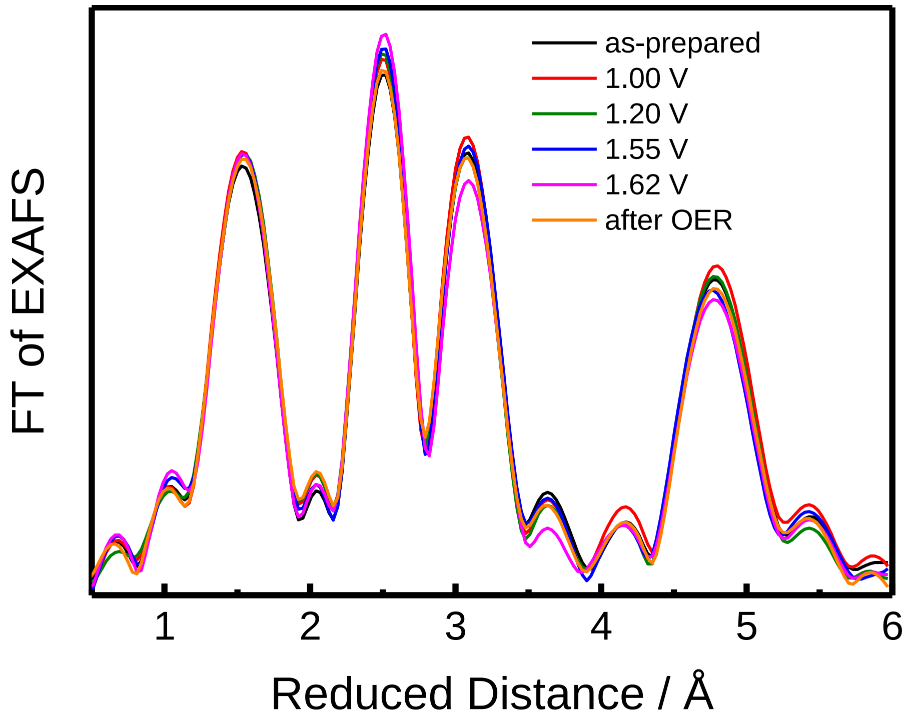

Supplementary Fig. 15: quasi-*in situ* FT of EXAFS spectra of Co_3O_4 films recorded at Co *K*-edge as function of catalyst state. Samples were freeze-quenched using liquid N_2 under electrochemical potential control after electrochemical conditioning for 15 min at the given potential in 0.1M KPi at pH 7.

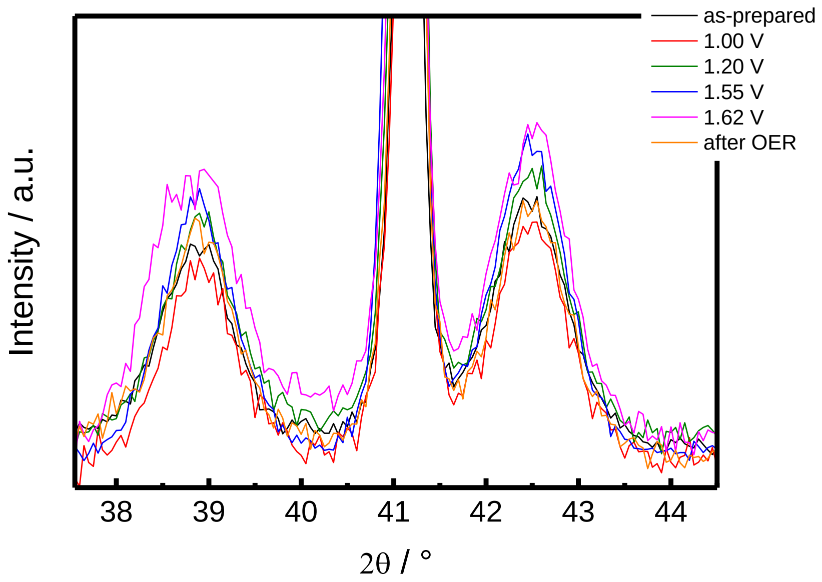

Supplementary Fig. 16: *in situ* X-ray diffraction patterns of Co_3O_4 catalyst films. The diffraction patterns were recorded using grazing-incident excitation at α=0.3° and 12 keV. The electrode potential was increased stepwise from 1.0 V to 1.62 V vs. RHE, the latter representing the catalytically-active state, in 0.1 M KPi at pH 7. The state after OER is a dry state for which the electrode was removed from electrolyte at 1.0 V rinsed with de-ionized water and dried in N_2 flow. Diffraction pattern were background corrected for better visualization.

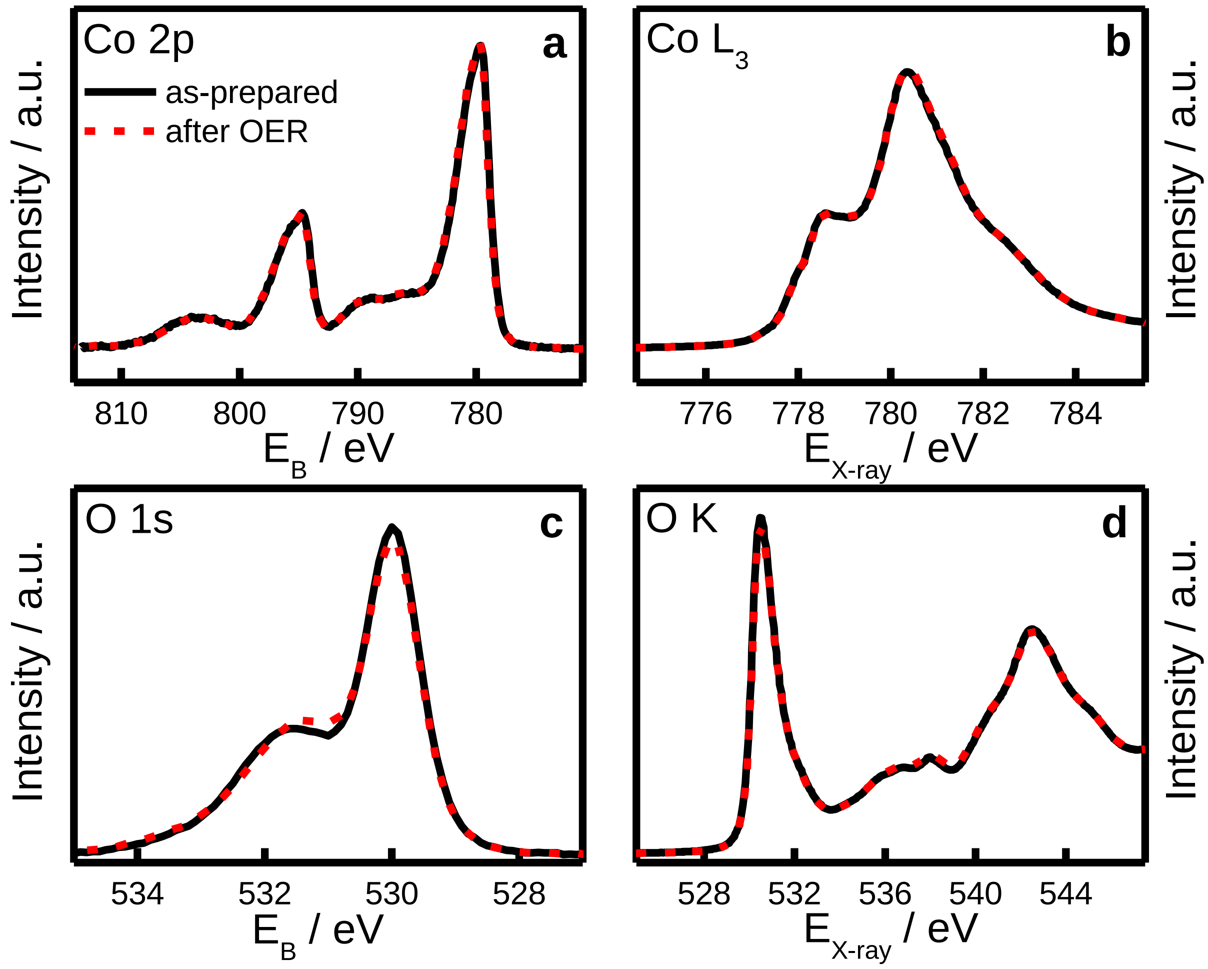

Supplementary Fig. 17: X-ray photoelectron spectra of Co 2p (a), O 1s (c) as well as XANES spectra recorded at Co L_3-(b) and O K-edge (d) of Co_3O_4 thin films prepared on GC. XPS spectra were recorded at photoelectron kinetic energy of 550 eV. XANES spectra were recorded in total-electron yield mode.

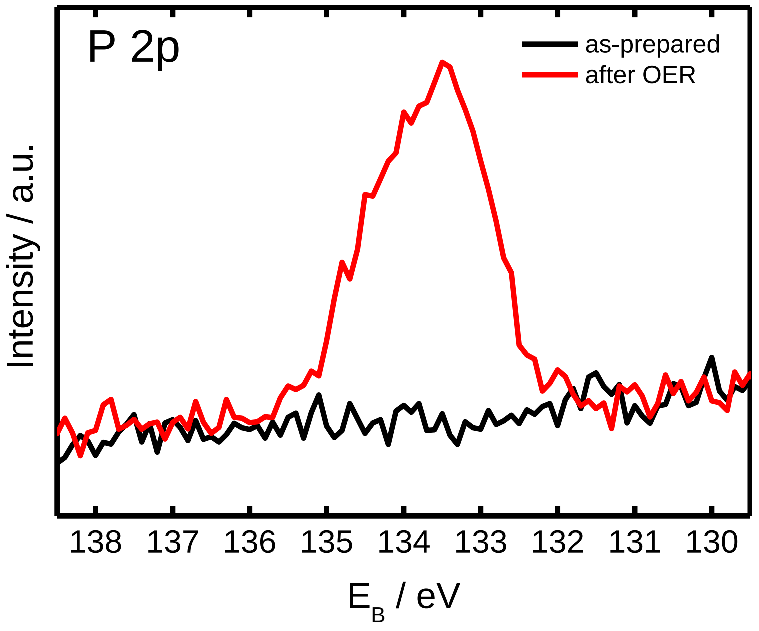

Supplementary Fig. 18: X-ray photoelectron spectra of P 2p recorded using as prepared Co_3O_4 thin films as prepared on glassy carbon and after 15 min of OER at 1.62 V in 0.1M KPi at pH 7. XPS spectra were recorded at photoelectron kinetic energy of 550 eV.

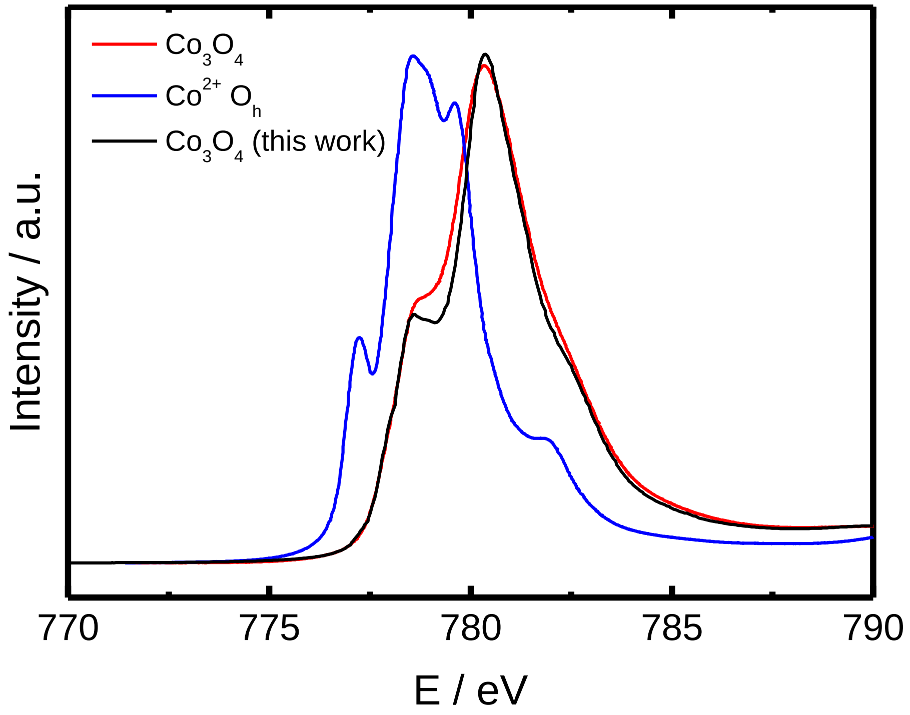

Supplementary Fig. 19: Co L_3-XANES spectra of Co_3O_4 thin films in the as-prepared state as well as of Co_3O_4 and Co^{2+} O_h references.[2] XANES spectra were recorded in total-electron yield mode.

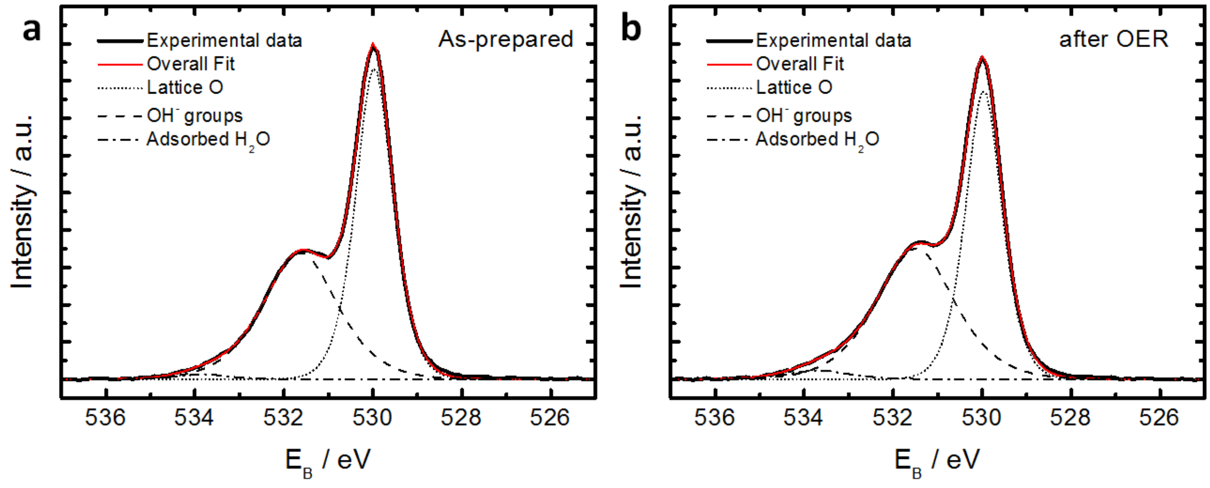

Supplementary Fig. 20: Fits of O 1s spectra of Co_3O_4 thin films as-prepared (a) and after oxygen evolution (b). XPS spectra were recorded at photoelectron kinetic energy of 550 eV. The fit of hydroxyl groups includes also contributions from oxygen species in phosphate molecules.

Supplementary Table 1: Fit results for Co_3O_4(511) and Co_3O_4(440) reflections of the *in situ* X-ray diffraction of thin films as function of catalyst state. Fitting was performed using pseudo-Voigt profiles. The error of the fit parameters represents one estimated standard deviation. The structural coherence length was determined from the integral breadth in the Scherrer equation. A shape factor of 0.89 was used.

Co_3O_4(511)	ASP	1.00V	1.20V	1.55V	1.62V	after OER
**2θ / °**	38.861±0.005	38.906±0.009	38.900±0.007	38.878±0.006	38.859±0.01	38.885±0.008
**d / Å**	1.5511±4E-4	1.5494±7E-4	1.5494±7E-4	1.5505±5E-4	1.5512±7E-4	1.5502±7E-4
**Height / cts**	123 ± 2	70 ± 2	73 ± 1	86 ± 1	99 ± 2	74 ± 2
**Area / cts·°**	2969 ± 56	1691 ± 50	1638 ± 42	1846 ±43	2767 ±76	1709 ±47
**FWHM / °**	0.936 ± 0.018	0.95 ± 0.02	0.91 ± 0.02	0.943 ± 0.019	1.13 ± 0.03	0.91 ± 0.02
**IB / °**	1.20 ± 0.04	1.20 ± 0.07	1.12 ± 0.04	1.07 ± 0.03	1.39± 0.06	1.15 ± 0.06
**CL_{IB} / nm**	4.62 ± 0.16	4.6 ± 0.2	4.97 ± 0.19	5.19 ± 0.18	3.99 ± 0.19	4.8 ± 0.2

Co_3O_4(440)	ASP	1.00V	1.20V	1.55V	1.62V	after OER
**2θ / °**	42.48±0.005	42.495±0.007	42.493±0.006	42.484±0.005	42.504±0.008	42.491±0.008
**d / Å**	1.4243±3E-4	1.4238±5E-4	1.4239±4E-4	1.4242±3E-4	1.4236±7E-4	1.4240±5E-4
**Height / cts**	147 ± 2	81 ± 1	88 ± 1	105 ± 1	112 ± 2	82 ± 1
**Area / cts·°**	3405 ± 61	17425 ± 43	1823± 39	2151 ± 41	2749 ± 75	1655 ± 42
**FWHM / °**	0.949 ± 0.015	0.91 ± 0.02	0.893 ± 0.018	0.910 ± 0.015	0.97 ± 0.02	0.92 ± 0.02
**IB / °**	1.15 ± 0.03	1.06 ± 0.03	1.03 ± 0.03	1.02 ± 0.02	1.22 ± 0.05	1.00 ± 0.03
**CL_{IB} / nm**	4.87 ± 0.15	5.30 ± 0.19	5.45 ± 0.17	5.51 ± 0.15	4.6 ± 0.2	5.5 ± 0.2

Supplementary Table 2: Simulation results for the fits of the k^3-weighted EXAFS spectra of Co_3O_4 thin films as a function of catalyst state. Fitting of the k^3-weighted data was done in k-space between 2.3 and 13 $Å^{-1}$. The amplitude-reduction factor S_0^2 was 0.8. A joint-fit approach was used, where the coordination numbers (*N*) were determined independently but the distances (*R*) and Debye-Waller factors (σ) had the same values for all samples; additionally, all Co shells had the same Debye-Waller factor. Fitting was performed using an in-house software (SimX) after calculation of the phase functions with the FEFF program[3,4] (version 8.4, self-consistent field option activated). The error ranges of the fit parameters were estimated from the covariance matrix of the fit and represent the 68% confidence intervals.[5]

Shell	Sample	ASP	1.00V	1.20V	1.55V	1.62V	after OER
**Co-O**	*N*	5.30	5.44	5.45	5.42	5.40	5.34
R = 1.908 +/- 0.001 Å	Error of *N*	0.2	0.2	0.2	0.2	0.2	0.2
σ = 0.046 +/- 0.002 Å							
**Co-Co**	*N*	4.63	4.73	4.76	4.83	5.01	4.61
R = 2.851 +/- 0.001 Å	Error of *N*	0.2	0.2	0.2	0.2	0.2	0.2
σ = 0.052 +/- 0.001 Å							
**Co-Co**	*N*	6.81	7.03	6.72	6.84	6.3	6.77
R = 3.357 +/- 0.001 Å	Error of *N*	0.3	0.3	0.3	0.3	0.3	0.3
σ = 0.052 +/- 0.001 Å							
**Co-Co**	*N*	7.73	7.37	7.7	7.98	7.27	7.29
R = 4.986 +/- 0.003 Å	Error of *N*	0.9	0.9	0.9	0.9	0.9	0.9
σ = 0.052 +/- 0.001 Å							
**Co-Co**	*N*	10.98	12.14	10.85	9.28	9.98	10.62
R = 5.315 +/- 0.002 Å	Error of *N*	1.1	1.1	1.1	1.1	1.1	1.1
σ = 0.052 +/- 0.001 Å							
**Co-Co-Co, multiple scattering from di-μ-oxo bridged Co on a straight line**	*N*	1.17	1.3	1.09	1.09	1.11	1.09
R = 5.703 +/- 0.002 Å	Error of *N*	0.4	0.4	0.4	0.4	0.4	0.4
σ = 0.052 +/- 0.001 Å							
	**Rf**	10.95	12.38	12.7	12.19	12.39	13.16

Supplementary Note 1

Supplementary Figure 17A and B show Co 2p XPS and the Co L_3 XANES spectra of Co_3O_4 thin films, respectively. The Co L_3 spectrum shows a main absorption peak at 780.3 eV accompanied by minor features at higher and lower X-ray energies. Comparison with reference spectra (Supplementary Fig. 19) shows that the near-surface of the as-prepared catalysts consists of mainly Co_3O_4 with a possible minor fraction of Co^{2+} O_h. The Co 2p spectrum of the Co_3O_4 shows two peaks at 779.7 and 794.7 eV arising from Co $2p_{3/2}$ and Co $2p_{1/2}$ states, respectively. The main $2p_{3/2}$ satellite at ~789 eV and the weaker satellite at ~786 eV are characteristic for a Co oxide containing mainly Co_3O_4 in the near-surface.

The O 1s spectrum (Supplementary Fig. 17C and 20) shows the presence of at least three different O species in the near-surface of the as-prepared state. Oxygen ions incorporated in the crystal lattice give rise to a peak at 530 eV, whereas the broad shoulder at ~531.5 eV is mainly caused by hydroxylated O.[6] Additionally, traces of adsorbed water are present leading to a contribution at ~533 eV.[6] The O K-edge absorption spectrum of the as-prepared state (Supplementary Fig. 17D) shows a strong absorption feature between 529 and 533 eV prior to the main O K absorption edge. This feature is caused by electron excitations from O 1s to hybridized states involving O 2p and Co 3d orbitals typical of Co^{3+} ions. The intensity of the pre-edge feature shows a strong hybridization of orbitals. The main absorption edge and the features above are assigned to electron transitions from the O 1s to hybridized orbitals of O 2p with Co 4s/4p states.[7,8] Thus, the near-surface of the as-prepared state consists of primarily Co_3O_4 with minor contribution of additional Co^{2+} O_h ions. The O/Co ratio (at photoelectron kinetic energy of 550 eV) is 1.48 and the fraction of lattice O is 54.4%.

In comparison to the as-prepared state of the Co_3O_4, the near-surface after OER contains a slightly lower fraction of lattice oxygen (49.5%) and exhibits a lower degree of O 2p-Co 3d orbital hybridization. This can be seen in the slight decrease of peak intensity at ~530 eV in the O 1s spectrum and in the pre-edge feature of O K-edge absorption spectrum. Additionally, phosphates are present in the near-surface after OER (P/Co = 0.05) which are most probably adsorbed at the Co_3O_4

surface. (Supplementary Fig. 18) The presence of the phosphate ions in the near-surface after OER also influences the O 1s spectrum (increased intensity at ~531 eV[6]) and the O K-edge absorption spectrum (additional feature between 535 and 540 eV).[9] We conclude that the increase of the O/Co ratio to 1.57 is thus primarily caused by the presence of the phosphate ions.

Supplementary References

1. McCrory, C. C. L., Jung, S., Peters, J. C., and Jaramillo, T. F., Benchmarking Heterogeneous Electrocatalysts for the Oxygen Evolution Reaction. *Journal of the American Chemical Society* **135** (45), 16977-16987 (2013).
2. Zafeiratos, S. et al., Methanol oxidation over model cobalt catalysts: Influence of the cobalt oxidation state on the reactivity. *Journal of Catalysis* **269** (2), 309-317 (2010).
3. Ankudinov, A. L., Ravel, B., Rehr, J. J., and Conradson, S. D., Real-space multiple-scattering calculation and interpretation of x-ray-absorption near-edge structure. *Physical Review B* **58** (12), 7565-7576 (1998).
4. Rehr, J. J. and Albers, R. C., Theoretical approaches to x-ray absorption fine structure. *Reviews of Modern Physics* **72** (3), 621-654 (2000).
5. Risch, M. et al., Nickel-oxido structure of a water-oxidizing catalyst film. *Chemical Communications* **47** (43), 11912-11914 (2011).
6. NIST X-ray Photoelectron Spectroscopy Database, Version 4.1 (National Institute of Standards and Technology, Gaithersburg, 2012); http://srdata.nist.gov/xps/.
7. Suntivich, J. et al., Estimating Hybridization of Transition Metal and Oxygen States in Perovskites from O K-edge X-ray Absorption Spectroscopy. *The Journal of Physical Chemistry C* **118** (4), 1856-1863 (2014).
8. de Groot, F. et al., Oxygen 1s x-ray-absorption edges of transition-metal oxides. *Physical Review B* **40** (8), 5715-5723 (1989).
9. Risch, M. et al., Atomic structure of cobalt-oxide nanoparticles active in light-driven catalysis of water oxidation. *International Journal of Hydrogen Energy* **37** (10), 8878-8888 (2012).
